# The Effect and Time Course of Prediction and Perceptual Load on Category-Based Attentional Orienting Across Color and Shape Dimensions

**DOI:** 10.3390/brainsci15111210

**Published:** 2025-11-09

**Authors:** Yunpeng Jiang, Tianyu Chen, Fangyuan Ou, Yun Wang, Ruixi Feng, Xia Wu, Lin Lin

**Affiliations:** 1 Faculty of Psychology, Tianjin Normal University, Tianjin 300387, China; jiangyp@tjnu.edu.cn (Y.J.); wuxia@tjnu.edu.cn (X.W.); 2 Key Research Base of Humanities and Social Sciences of the Ministry of Education, Academy of Psychology and Behavior, Tianjin 300387, China; 3 Center of Collaborative Innovation for Assessment and Promotion of Mental Health, Tianjin 300387, China

**Keywords:** category-based attentional orienting, uncertainty, perceptual load, prediction, N2pc

## Abstract

**Objectives:** This study investigated the temporal dynamics of category-based attentional orienting (CAO) under the influences of prediction (top-down) and perceptual load (bottom-up) across color and shape dimensions, combining behavioral and event-related potential (ERP) measures. **Methods:** Across two experiments, we manipulated predictive validity and perceptual load during a visual search for category-defined targets. **Results:** The results revealed a critical dimension-specific effect of prediction: invalid predictions elicited a larger N2pc component (indexing attentional selection) for shape-defined targets, but not color-defined targets, indicating that shape CAO relies more heavily on predictive information during early processing. At the behavioral level, a combined analysis of the two experiments revealed an interaction between prediction and perceptual load on accuracy, suggesting their integration can occur at later stages. **Conclusions:** These findings demonstrate that prediction and perceptual load exhibit distinct temporal profiles, primarily independently modulating early attentional orienting, with their interactive effects on behavior being more nuanced and dimension-dependent. This study elucidates the distinct temporal and dimensional mechanisms through which top-down and bottom-up sources of uncertainty shape attentional orienting to categories.

## 1. Introduction

Individuals allocate attentional resources to target-relevant locations in order to adapt to the complex external environment. Attentional orienting plays an important role in this process, which refers to the mental process of selecting a target and shifting attention to relevant stimuli among complex external stimuli [1]. While previous research has focused on orientation to single and basic features (e.g., color [2]), real-world stimuli are more complex and require attention to multiple features. Category-based attentional orienting (CAO) refers to the process of selectively attending to stimuli based on their category membership [3,4,5,6,7]. Categories are sets of features that reflect the processing of complex objects. CAO allows attention to meaningful real-world stimuli, like road signs containing informative versus prohibitive categories. CAO is guided by categorical templates maintained in working memory that orient attention toward category-matching stimuli [8]. Elucidating the mechanisms underlying CAO can provide insight into how people efficiently orient attention to complex, multi-featured stimuli, with implications for improving visual search and task performance.

Stimulus uncertainty affects individuals’ perception and attention, including CAO. Uncertainty is determined by the complexity of the sensory information and the size of the mental computation [9]. Prediction and perceptual load are two manifestations of uncertainty. Prediction reflects a state of readiness to perceive upcoming information based on prior background information [10]. Perceptual load refers to the quantity of task-relevant sensory information that must be processed simultaneously during an attention-demanding activity [11]. Although some research progress has been made regarding the mechanisms through which these two factors affect CAO, there are still some unresolved issues.

Prediction can be formed by learning statistical regularities between cues and targets, subsequently applying this learned relationship to generate appropriate responses to the targets [12,13]. In studies employing cueing paradigms, some researchers used auditory or color cues and manipulated predictability by controlling the ratio of valid to invalid cues [14,15]. This manipulation induced predictions, and participants were required to judge the target’s orientation. These studies found that performance was better under valid cue conditions than under invalid cue conditions, and this behavioral advantage increased with the predictability of the cues. Esterman and Yantis (2010) investigated the effect of predictive cues on stimuli from different categories (e.g., houses and faces), finding that valid cues facilitated the perceptual processing of information across categories [16]. However, searching for a categorical target, as opposed to a single-feature target, necessitates simultaneously monitoring multiple objects, which increases the visual processing load. Furthermore, it remains unclear whether the facilitative effect of prediction on the perceptual representation of category information can subsequently influence attentional orienting. Therefore, the impact of prediction on CAO warrants further investigation.

Perceptual load in experimental tasks can be systematically modulated by controlling the degree of homogeneity among distractors presented simultaneously with the target. When distractors are homogeneous, perceptual load is low, attentional resources are concentrated, and target processing is efficient; whereas when distractors are heterogeneous, perceptual load increases, attentional resources disperse, and efficiency decreases [17,18]. Yang and Zelinsky (2009) asked participants to search for teddy bears among different daily necessities [19]. The characteristics of teddy bears varied but belonged to the same category. The distractor necessities changed randomly. This requires excluding complex, variable distractors and attending to multi-featured teddy bears. Therefore, searching for teddy bears involves attentional orienting to category information with higher perceptual load.

Although both prediction and perceptual load impact CAO individually, their combined influence remains unclear. Neuroimaging studies suggest the frontoparietal network is involved in both types of attentional processes [20], while behavioral evidence indicates they can operate independently [21]. Critically, the nature of their interaction—specifically, whether and how prediction and perceptual load interact across the time course of CAO, and whether this interplay differs for categories defined by different feature dimensions—remains a central and unexplored question. This gap limits our understanding of how multiple sources of uncertainty are integrated during complex visual search.

The potential dimension-specificity of these effects presents a compelling lens through which to address this question. During attentional orienting, allocating attention to target categories can be influenced by different feature dimensions [7]. The dimensional weighting theory proposes that feature dimensions differ in weight during perceptual and executive control processing [22]. Color and shape are two feature dimensions processed through multiple brain regions (V1/V2-V4-TEO/IT) in the ventral pathway, including the occipital to temporal lobes. These regions can form attentional templates to guide attention [7,23,24]. Compared to shape, the color dimension shows processing advantages [6,25]. Wu et al. (2020) used both color and shape as category targets, finding that they elicited independent attentional orienting at the early stage, with color showing stronger effects [7]. It can be seen that category information also produces different weights of process due to different dimensions [26]. Therefore, systematically comparing how prediction and perceptual load influence color and shape CAO provides a powerful approach to elucidate the fundamental characteristics of CAO—specifically, whether it is governed by a universal mechanism or is dimension-specific. Investigating the distinct and combined effects of uncertainty across dimensions can establish a refined, dimension-specific framework for attentional guidance.

To examine the effects of prediction and perceptual load on CAO across the color and shape dimensions, we conducted two experiments that combined an associative learning task with a visual search task while recording event-related potentials (ERPs) [27]. In Experiment 1, targets were defined by color category (warm/cool), while in Experiment 2, they were defined by shape category (“X”/“O”). Prediction was manipulated through varying auditory cue–target location validity, and perceptual load was manipulated by altering distractor homogeneity. We focused our ERP analyses on the P1 and N2pc components to dissect the temporal dynamics of early visual processing [28] and spatial attentional selection [29,30], respectively. We expected both factors to influence behavioral performance. Our central hypothesis, grounded in dimensional differences in salience, was that the neural mechanisms of early attentional selection would be dimension-specific: shape CAO would critically rely on top-down prediction (as reflected in the N2pc), whereas color CAO, due to its high salience, would be less dependent on such predictive cues.

## 2. Experiment 1

### 2.1. Method

#### 2.1.1. Participants

The required sample size was estimated using G*Power 3.1.9.4 [31]. Assuming a medium effect size (f = 0.25), α = 0.05, and power (1 − β) = 0.80, the analysis indicated a minimum of 24 participants. Accordingly, thirty-three students from Tianjin Normal University were recruited via campus posters and WeChat groups. Participants were compensated with a small monetary reward. All participants reported normal or corrected-to-normal visual acuity and color vision, no history of mental illness, and were right-handed. Four participants were excluded due to low accuracy or excessive EEG artifacts, leaving 27 participants (17 females; M = 20.60 ± 1.94 years) for the final analysis. The study was approved by the Ethics Committee of Tianjin Normal University, and written informed consent was obtained from all participants.

#### 2.1.2. Design and Procedure

The experiment was a 2 (prediction: invalid, valid) × 2 (perceptual load: low, high) within-subjects design that combined an associative learning task (using auditory cues with 80% validity to induce predictions about target location) with a visual search task (manipulating distractor homogeneity—low load: identical distractors; high load: variable distractors within the same category—to vary perceptual load).

**Figure 1:**
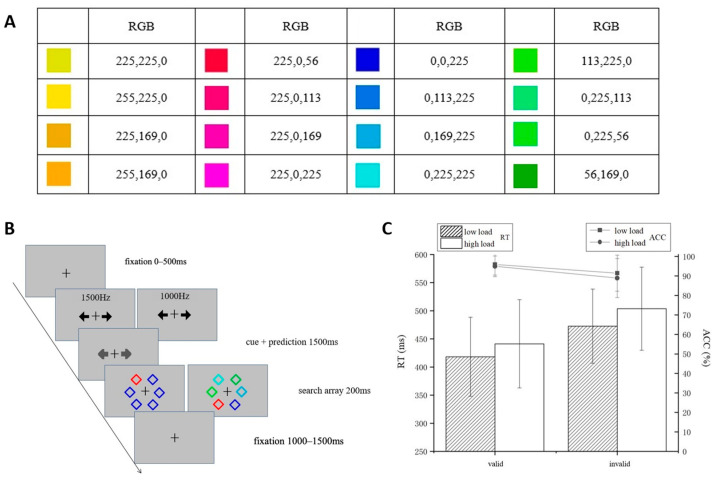
Experiment 1. (**A**) The colors used in Experiment 1 and their corresponding RGB values. The two columns on the left are warm colors, and the two columns on the right are cool colors. (**B**) Experimental procedure. Participants were asked to make judgments of the location of the warm color (left and right) under low or high perceptual load conditions. The cue (pure tones at 1500 Hz or 1000 Hz) was presented before the search display, and there is a correlation between the cue and the position of the warm color. (**C**) Reaction time (bar: RTs/ms) and accuracy (line: ACC/%) under different conditions.

The experiments were conducted in a soundproof, electromagnetic shielded room. All stimuli were displayed on a 19-inch CRT monitor, which was configured at a screen resolution of 1024 × 768 pixels with a refresh rate of 75 Hz. Participants were positioned at a distance of 60 cm from the display. The targets were defined as color categories, i.e., eight warm colors and eight cool colors, as shown in Figure 1A. E-Prime 3.0 software was used to present the experiments and collect behavioral data. Participants were trained to categorize warm and cool colors with 100% accuracy before entering the formal experiment. During practice, participants were asked to categorize colors as warm or cool via key press. Feedback was given following each response.

The procedure of the formal experiment was as follows (Figure 1B): Each trial commenced with a cross displayed for a variable duration of 0–500 ms on a gray background (RGB: 192, 192, 192). Subsequently, participants were presented with an auditory cue—either a high-frequency (1500 Hz) or low-frequency (1000 Hz) tone. Based on this auditory cue, participants were required to predict the target’s upcoming location within 1500 ms by pressing either the “a” key (indicating left side prediction) or the “s” key (indicating right side prediction). Visual feedback was provided as the two on-screen arrows changed from black to gray upon key press. The association between auditory cues and target positions (high tone-left, high tone-right, low tone-left, and low tone-right) was counterbalanced across blocks. The predictive relationship between cues and targets maintained 80% validity (targets appearing at the cue-indicated location) and 20% invalidity (targets appearing at the opposite location). (The cue validity here is different from the spatial cueing paradigm. First, the location relationship between the cue and the target is not fixed, only the probability is different; second, the cue itself has no spatial information, avoiding the influence of spatial attention.)

Following the prediction phase, a search display appeared for 200 ms, consisting of a single target accompanied by five distractors. The stimuli (each subtending 0.5° × 0.5°) were randomly distributed among six possible locations arranged in a circular pattern around the fixation point, at a radial distance of 3° from the center. Target category assignment (warm or cool) was counterbalanced across participants. In each display, one stimulus matched the defined target color while the remaining five distractors belonged to the opposing category. Participants were required to indicate the target’s location by pressing “k” to indicate left or “l” to indicate right within a response window of 1–1.5 s. Perceptual load was manipulated through distractor homogeneity: in the low-load condition, all distractors were identical, whereas in the high-load condition, distractors varied while remaining within the same category. Each trial lasted 3200 ms.

Experiment 1 included 24 practice trials and 4 blocks of 120 trials each (96 valid trials, 24 invalid per block) in the formal experiment. The whole experiment lasted approximately 30 min.

#### 2.1.3. ERP Recording and Analysis

EEG data were acquired using a CURRY 8 system from NeuroScan, El Paso, TX, USA, and a 10–20 international system extended with 64 conductive electrode caps. EEG data were recorded continuously with a sampling rate of 1000 Hz. Vertical electrooculography (vEOG) and horizontal electrooculography (hEOG) were recorded. M1 and M2 were used as reference electrodes. The electrical resistance of all electrodes was kept below 5 kΩ.

Data were analyzed offline using the EEGLAB toolbox [32] and custom-written MATLAB (R2022b) functions. The raw data were filtered with a 0.5 Hz high-pass and 40 Hz low-pass filter. ERPs were extracted in epochs from −200 and 800 ms relative to stimulus onset. Baseline correction was performed by subtracting the mean amplitude of each channel from a 200 ms window prior to the search array onset. Artifact rejection was conducted using independent component analysis (ICA) to remove ocular artifacts, followed by automatic rejection of epochs exceeding ±100 μV amplitude thresholds and manual inspection for the remaining artifacts. An average of 10.9% (range = 2.5–18.3%) of trials were rejected, while in Experiment 2, an average of 9.2% (range = 1.7–21.7%) of trials were rejected. P1 and N2pc components were quantified at lateral occipital sites PO7/PO8. Ipsilateral and contralateral waveforms were computed relative to the target side. Difference waves were calculated as contralateral minus ipsilateral. According to the point-by-point t-test, the time windows in which the components were more conducive to statistical analysis were selected. The point-by-point *t*-test involved conducting one-sample t-tests at each time point (from 0 to 500 ms post-stimulus) to identify clusters of consecutive time points (minimum 20 ms) where the waveform significantly deviated from zero (*p* < 0.05). P1 was defined as the mean amplitude from 90 to 150 ms post-stimulus. N2pc was defined as the mean amplitude from 230 to 290 ms (Experiment 1) and 220–280 ms (Experiment 2) based on previous studies.

### 2.2. Results

#### 2.2.1. Behavioral Data

A 2 (prediction: invalid, valid) × 2 (perceptual load: low, high) repeated-measures ANOVA was performed on accuracy (ACC) and response time (RT). Figure 1C depicts the ACC and RT in line and bar, respectively. Simple effects analyses were corrected using the Bonferroni method.

The results for ACC found that the main effect of prediction was significant, *F*(1, 26) = 16.95, *p* < 0.001, η^2^ = 0.39, and the ACC of the valid condition (95.4 ± 5.0%) was significantly higher than that of the invalid condition (90.2 ± 10%), indicating that effective associated learning was performed. The main effect of perceptual load was significant, *F*(1, 26) = 6.71, *p* = 0.016, η^2^ = 0.21, with a significantly higher ACC for low load (93.7 ± 7%) than for high load (91.9 ± 7%). The interaction between prediction and perceptual load was not significant, *F*(1, 26) = 2.26, *p* = 0.15, η^2^ = 0.08, BF = 1.68.

The results for RT found that the main effect of prediction was significant, *F*(1, 26) = 62.57, *p <* 0.001, η^2^ = 0.71, and the valid condition (429.76 ± 74.28 ms) was significantly shorter than the invalid condition (488.19 ± 69.93 ms). The main effect of perceptual load was significant, *F*(1, 26) = 23.71, *p* < 0.001, η^2^ = 0.48, and the RT for low load (445.44 ± 68.10 ms) was significantly shorter than that for high load (472.51 ± 76.12 ms). The interaction between prediction and perceptual load was not significant, *F*(1, 26) = 1.265, *p* = 0.271, η^2^ = 0.046, BF = 2.73.

#### 2.2.2. ERP Data

**Figure 2:**
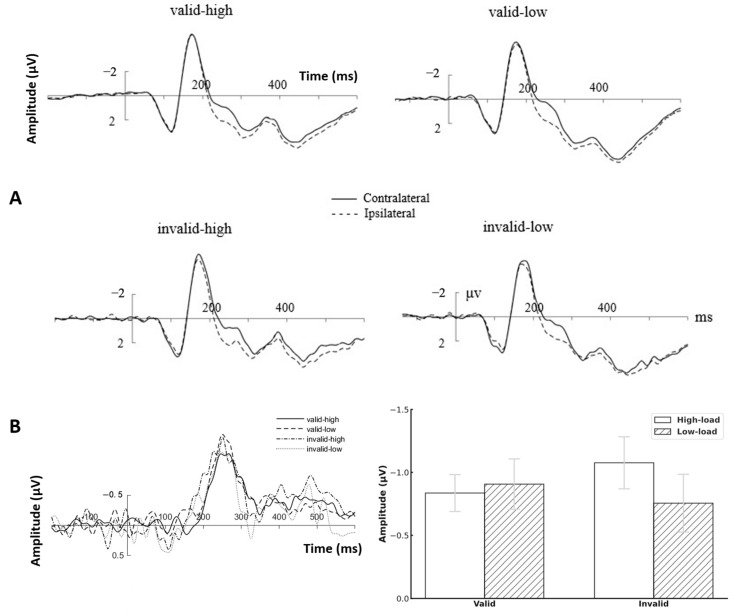
ERPs results in Experiment 1. (**A**) Contralateral and ipsilateral waveforms induced by PO7/PO8 under different conditions. (**B**) Left: Difference waveforms obtained by subtracting the ipsilateral waveforms from the contralateral waveforms. Right: The mean amplitudes of difference waves of N2pc under each condition.

Figure 2A shows the contralateral and ipsilateral waveforms evoked by PO7/PO8 under different conditions. A 2 (prediction: invalid, valid) × 2 (perceptual load: low, high) repeated-measures ANOVA was performed on the lateralized P1 amplitude. The main effect of prediction was significant, *F*(1, 26) = 4.28, *p* = 0.049, η^2^ = 0.14, and the amplitude of the invalid condition was significantly larger than that of the valid condition. The main effect of perceptual load was not significant, *F*(1, 26) *=* 0.95, *p =* 0.339, η^2^ = 0.035, BF = 3.12. The interaction between prediction and perceptual load was not significant, *F*(1, 26) = 0.038, *p* = 0.566, η^2^ = 0.013, BF = 5.09.

A 2 (laterality: contralateral, ipsilateral) × 2 (prediction: invalid, valid) × 2 (perceptual load: low, high) repeated-measures ANOVA was performed on the N2pc amplitude. The results showed that the main effect of laterality was significant, *F*(1, 26) = 49.21, *p* < 0.001, η^2^ = 0.65, with the contralateral waveforms being significantly more negative than the ipsilateral waveforms, indicating the presence of a robust N2pc component.

The difference waves between contralateral and ipsilateral (Figure 2B) were further subjected to a 2 (prediction: invalid, valid) × 2 (perceptual load: low load, high load) repeated-measures ANOVA. The results showed that the main effect of perceptual load was not significant, *F*(1, 26) = 0.09, *p* = 0.766, η^2^ = 0.004, BF = 4.95. The main effect of prediction was not significant, *F*(1, 26) = 0.09, *p =* 0.766, η^2^ < 0.001, BF = 3.57. The interaction between prediction and perceptual load was not significant, *F*(1, 26) = 1.065, *p* = 0.312, η^2^ = 0.066, BF = 3.02.

## 3. Experiment 2

### 3.1. Method

#### 3.1.1. Participants

The required sample size was estimated using G*Power 3.1.9.4 [31]. Assuming a medium effect size (f = 0.25), α = 0.05, and power (1 − β) = 0.80, the analysis indicated a minimum of 24 participants. Accordingly, thirty students from Tianjin Normal University were recruited via campus posters and WeChat groups. Participants were compensated with a small monetary reward. All had normal or corrected-to-normal visual and color vision, no history of mental illness, and were right-handed. Three participants were excluded due to excessive artifacts, resulting in a final sample of 27 (13 females; M = 21.96 ± 2.08 years). The study was approved by the Ethics Committee of Tianjin Normal University, and all participants provided written informed consent prior to the experiment.

#### 3.1.2. Design and Procedure

**Figure 3:**
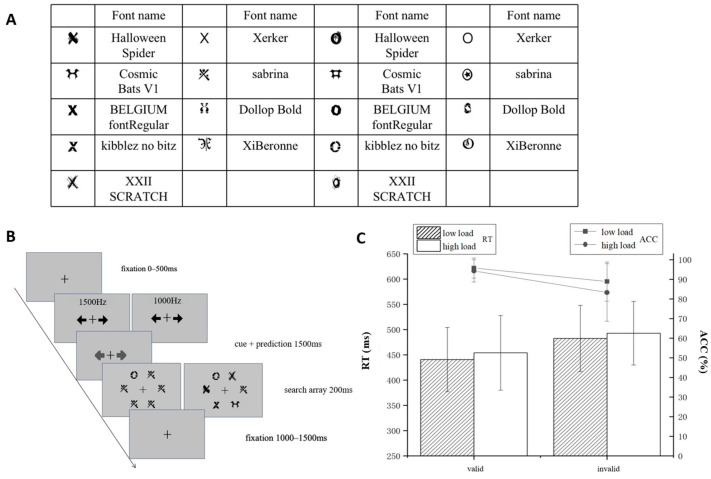
Experiment 2. (**A**) Materials used in the experiment. The left two columns are the fonts used for the “X” shape, and the right two columns are the fonts used for the “O” shape. (**B**) Experimental procedure. Participants were asked to make judgments of the location of the “O” shape (left and right) under low or high perceptual load conditions. The cue (pure tones at 1500 Hz or 1000 Hz) was presented before the search display, and there is a correlation between the cue and the position of the “O” shape. (**C**) Reaction time (bar: RT/ms) and accuracy (line: ACC/%) under different conditions.

Experiment 2 defined the targets as shape categories (“X” shape, “O” shape), consisting of different fonts (Figure 3A). The design and procedure (Figure 3B) were the same as Experiment 1.

### 3.2. Results

#### 3.2.1. Behavioral Data

A 2 (prediction: invalid, valid) × 2 (perceptual load: low, high) repeated-measures ANOVA was performed for ACC and RT. Figure 3C depicts the ACC and RT in line and bar, respectively. Simple effects analyses were corrected using the Bonferroni method.

The results of ACC found that the main effect of prediction was significant, *F*(1, 26) = 19.64, *p <* 0.001, η^2^ = 0.43, and the valid condition (95.1 ± 5%) was significantly higher than the invalid condition (86.1 ± 12%), indicating that effective associated learning was performed. The main effect of perceptual load was significant, *F*(1, 26) = 17.41, *p* < 0.001, η^2^ = 0.40. ACC was higher under low load (92.4 ± 8%) than under high load (88.8 ± 10%). The interaction between the two factors was significant, *F*(1, 26) = 5.591, *p =* 0.026, η^2^ = 0.18. Simple effects analysis revealed that the ACC was lower for high load than for low load, regardless of the validity of the prediction. The ACC of invalid prediction was lower than that of valid prediction for both high load [*F*(1, 26) = 18.004, *p <* 0.001, η^2^ = 0.41] and low load [*F*(1, 26) = 16.284, *p* < 0.001, η^2^ = 0.39].

The results for RT revealed a significant main effect of prediction, *F*(1, 26) = 49.246, *p <* 0.001, η^2^ = 0.65, and the RT was significantly shorter for the valid condition (456.28 ± 72.62 ms) than in invalid expectations (518.66 ± 71.72 ms). The main effect of perceptual load was also significant, *F*(1, 26) = 19.317, *p* < 0.001, η^2^ = 0.43. The RT for low load (479.29 ± 68.55 ms) was significantly shorter than that for high load (495.65 ± 75.79 ms). The interaction between prediction and perceptual load was not significant, *F*(1, 26) = 2.207, *p* = 0.149, η^2^ = 0.078, BF = 1.73.

#### 3.2.2. ERP Data

**Figure 4:**
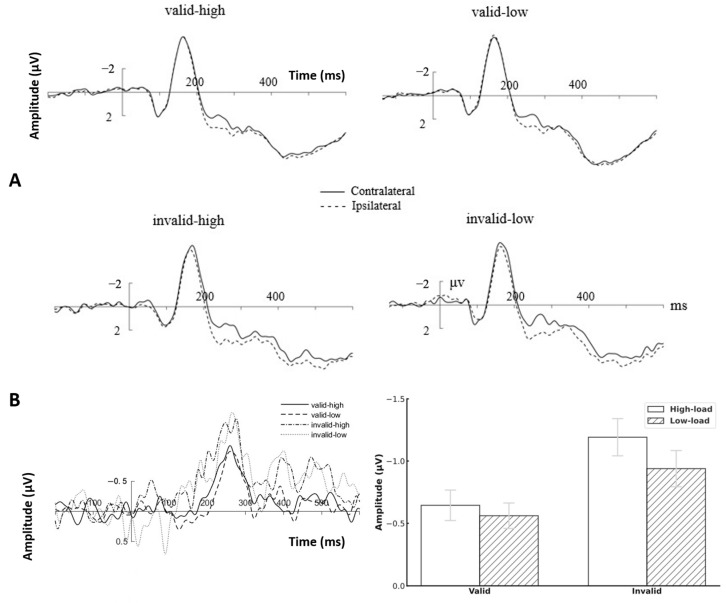
ERPs results in Experiment 2. (**A**) Contralateral and ipsilateral waveforms induced by PO7/PO8 under different conditions. (**B**) Left: Difference waveforms obtained by subtracting the ipsilateral waveforms from the contralateral waveforms. Right: The mean amplitudes of difference waves of N2pc under each condition.

Figure 4A shows the contralateral and ipsilateral waveforms evoked by PO7/PO8 under different conditions in Experiment 2. A 2 (prediction: invalid, valid) × 2 (perceptual load: low, high) repeated-measures ANOVA was performed on the P1 amplitude. The main effect of prediction was not significant, *F*(1, 26) *=* 2.223, *p =* 0.148, η^2^ = 0.079, BF = 1.72. The main effect of perceptual load was not significant, *F*(1, 26) = 1.674, *p =* 0.207, η^2^ = 0.060, BF = 2.24. The interaction between the two factors was not significant, *F*(1, 26) = 0.05, *p* = 0.825, η^2^ = 0.002, BF = 5.10.

A 2 (laterality: contralateral, ipsilateral) × 2 (prediction: invalid, valid) × 2 (perceptual load: low, high) repeated-measures ANOVA was performed on the N2pc amplitude. The results showed that the main effect of laterality was significant, *F*(1, 26) = 100.344, *p* < 0.001, η^2^ = 0.79, and a significantly more negative potential was observed at contralateral sites compared to ipsilateral sites, reflecting a significant N2pc component. The interaction between validity and laterality was significant, *F*(1, 26) = 13.935, *p* < 0.001, η^2^ = 0.35. Simple effects analysis revealed that the amplitude of the ipsilateral was significantly larger than that of the contralateral, both under the valid condition [*F*(1, 26) *=* 61.973, *p* < 0.001, η^2^ = 0.70] and the invalid condition [*F*(1, 26) = 103.516, *p* < 0.001, η^2^ = 0.80].

The difference waves of contralateral and ipsilateral (Figure 4B) were further subjected to a 2 (prediction: invalid, valid) × 2 (perceptual load: low, high) repeated-measures ANOVA. The results showed that the main effect of prediction was significant, *F*(1, 26) = 14.016, *p* = 0.001, η^2^ = 0.35. Valid conditions elicited significantly larger (more negative) N2pc amplitudes than invalid conditions, which indicated that individuals needed to devote more attentional resources when the prediction was invalid. The main effect of perceptual load was not significant, *F*(1, 26) = 0.073, *p =* 0.789, η^2^ = 0.436, BF = 5.00. The interaction between prediction and perceptual load was not significant, *F*(1, 26) = 0.008, *p* = 0.929, η^2^ < 0.001, BF = 5.20.

## 4. Overall Comparison

To examine how prediction and perceptual load affect category-based attentional orienting (CAO) across the dimensions of color and shape, we conducted a three-way repeated-measures ANOVA with prediction (invalid vs. valid), perceptual load (low vs. high), and category dimension (shape vs. color) as factors, analyzing both behavioral measures and N2pc components. The prediction and perceptual load were within-subject variables, and the category dimensions were between-subject variables.

The ACC results found a significant main effect of prediction, *F*(1, 52) = 35.347, *p* < 0.001, η^2^ *=* 0.41, and the ACC of the valid condition (95.2 ± 5.32%) was significantly higher than that of the invalid condition (88.1 ± 11.36%). The main effect of perceptual load was significant, *F*(1, 52) = 23.815, *p* < 0.001, η^2^ = 0.31, and the ACC was significantly higher for low load (93.0 ± 10.59%) than for high load (90.4% ± 8.18). The interaction between prediction and perceptual load was significant, *F*(1, 52) = 7.838, *p =* 0.007, η^2^ = 0.13. Simple effects analysis revealed that whether the prediction was valid [*F*(1, 52) = 14.328, *p* < 0.001] or not [*F*(1, 52) = 16.66, *p* < 0.001], the low load ACC was higher than that of the high load. The ACC of valid prediction was greater than that of invalid prediction for both high [*F*(1, 52) = 31.654, *p* < 0.001] and low [*F*(1, 52) = 30.303, *p <* 0.001] loads. This interaction indicates that prediction and perceptual load integrate at the behavioral level to influence accuracy, with reduced uncertainty (valid predictions and low load) yielding the highest performance across dimensions.

The RT results showed that the main effect of prediction was significant, *F*(1, 52) = 109.26, *p* < 0.001, η^2^ = 0.68, and the valid condition (443.02 ± 73.95 ms) was significantly shorter than that of the invalid condition (503.43 ± 72.49 ms). The main effect of perceptual load was significant, *F*(1, 52) = 42.137, *p <* 0.001, η^2^ = 0.45. The RT was significantly shorter for the low load condition (462 ± 74.72 ms) than the high load condition (484.08 ± 82.03 ms). These main effects confirm the independent contributions of prediction and perceptual load to response speed, without dimension-specific differences.

N2pc results found a significant main effect of prediction, *F*(1, 52) = 5.992, *p =* 0.018, η^2^ = 0.10, with larger N2pc amplitudes observed in the invalid condition compared to the valid condition. A significant interaction emerged between prediction and category dimension, *F*(1, 52) *=* 6.803, *p =* 0.012, η^2^ = 0.12. Simple effects analysis found that while N2pc amplitudes did not differ significantly between valid and invalid conditions in the color dimension, *F*(1, 52) = 0.013, *p =* 0.910, η^2^ < 0.001, BF = 6.69. A significant difference was observed in the shape dimension, *F*(1, 52) = 12.783, *p =* 0.001, η^2^ = 0.20, where invalid conditions elicited significantly larger N2pc amplitudes than valid conditions. The prediction-by-dimension interaction highlights dimension-specific neural mechanisms: invalid predictions demand greater attentional resources (larger N2pc) for shape but not color CAO.

## 5. Discussion

This study examined the influences of prediction and perceptual load on category-based attentional orienting (CAO) across color and shape dimensions using associative learning and visual search paradigms. N2pc results showed a significant interaction between prediction and category dimension: invalid predictions elicited more negative N2pc amplitudes (indicating greater attentional resource allocation) for shape-defined targets but not for color-defined targets. Behavioral data revealed a significant interaction between prediction and perceptual load on accuracy, with higher performance under reduced uncertainty (valid cues and low load). These findings indicate that prediction and perceptual load independently modulate early CAO in a dimension-specific manner (as indexed by N2pc), but interact at later response stages. Specifically, shape CAO relies more on top-down prediction during early processing, whereas color CAO is less prediction-dependent, likely due to its inherent salience. This elucidates the distinct temporal mechanisms of top-down and bottom-up uncertainty in CAO, offering insights for enhancing visual search efficiency in real-world scenarios by leveraging dimension-specific cues.

Top-down prediction influenced CAO across both dimensions, as evidenced by faster RTs and higher accuracy in valid conditions. However, its neural impact—manifested as smaller N2pc amplitudes in valid trials—was specific to the shape dimension, suggesting that valid cues facilitate early attentional selection for shapes by reducing resource demands, while color CAO shows no such modulation. This inference of reduced resource demands under valid predictions is directly based on the observed attenuation of the N2pc amplitude. In invalid trials, greater attentional resources are required to resolve conflicts, leading to more negative N2pc (for shapes) and poorer behavioral performance. This may arise from predictive cues preemptively activating category-relevant perceptual representations in V1, thereby sharpening processing and reducing neural activity [15,16]. In contrast, invalid cues necessitate heightened activity to overcome mismatches [33]. While prior work has shown prediction’s effects on low-level features [34] and complex categories like faces [16,35], our findings extend this to CAO, highlighting how category templates in working memory enable efficient use of anticipatory information for multi-feature targets [8]. This dimension-specific reliance aligns with subregional preferences in the ventral visual cortex (color: right hV4 [36]; shape: right VO1/VO2 [37]) and evidence that color elicits easier attentional capture due to higher salience [6,7,25], enabling rapid orienting independent of prediction for color while enhancing efficiency via prediction for shape.

Bottom-up perceptual load also modulated CAO across both dimensions, with more efficient orienting (shorter RTs, higher accuracy) under low-load conditions. This can be attributed to reduced perceptual differences between targets and distractors in low-load trials, where homogeneous distractors allow focused resource allocation [17]. In our paradigm, targets and distractors shared dimensions but differed in categories (e.g., warm vs. cool colors), with intra-category variability amplifying uncertainty in high-load conditions, mimicking real-world complexity [19]. High-load heterogeneity dispersed attention across multiple comparisons, diminishing efficiency, whereas low-load homogeneity enhanced target salience and bottom-up capture [38]. Thus, our findings extend perceptual load theory to categorical search, highlighting its role in modulating target-distractor discrimination.

The observed interaction between prediction and perceptual load on accuracy suggests that CAO involves the integration of top-down and bottom-up information. Notably, while N2pc (indexing early spatial selection [30,39]) showed no interaction—consistent with independent early effects—behavioral measures did, indicating interactive effects during later response selection. This dimension-specific pattern in N2pc further underscores that integration may vary: for shapes, early independence gives way to late synergy, whereas color’s salience minimizes early prediction reliance. This aligns with [40], who found bottom-up dominance early in single-feature search, overridden by top-down processes later [40]. To our knowledge, this is the first study extending this to CAO, where category templates guide search [41]. The lack of N2pc interaction supports early independence [21], potentially linked to distinct activations in primary visual and premotor cortices [42]. In contrast, late-stage integration, possibly via frontoparietal networks [20], enhances performance under combined low uncertainty. Overall, CAO’s complexity demands greater coordination of uncertainties compared to simple features.

Despite these contributions, limitations exist. We examined only two feature dimensions (color: warm/cool; shape: X/O), which may not capture the full complexity of real-world stimuli and dynamic environments. The sample was limited to young university students, potentially reducing generalizability. Future studies could incorporate neuroimaging (e.g., fMRI) to explore neural networks beyond ERPs, or test CAO in naturalistic settings (e.g., augmented reality tasks) with diverse populations. This may reveal broader applications, such as optimizing visual interfaces in high-uncertainty contexts like driving or medical imaging, to enhance efficiency.

## 6. Conclusions

In conclusion, this study delineates the temporal and dimensional architecture of category-based attentional orienting (CAO). Our findings provide a refined theoretical framework for understanding how uncertainty shapes attention, establishing a fundamental dissociation: the reliance on top-down prediction during early attentional selection is dimension-specific, being critical for shape but not color CAO, as revealed by the N2pc. Furthermore, the temporal profiles of top-down and bottom-up uncertainty are dissociable: they predominantly act as independent factors during early orienting, while their combined influence on behavioral outcomes emerges under specific conditions and dimensions. This work reveals that CAO is not a unitary process but is governed by dissociable interactions between feature dimensions and sources of uncertainty across time, advancing our theoretical understanding of attentional control in complex environments.

## Data Availability

The original data presented in the study are openly available in the Open Science Framework (OSF) at https://osf.io/j2trx/ (26 September 2025).
